# Cognitive-Enhancing, *Ex Vivo* Antilipid Peroxidation and Qualitative Phytochemical Evaluation of the Aqueous and Methanolic Stem Bark Extracts of *Lonchocarpus eriocalyx* (Harms.)

**DOI:** 10.1155/2020/8819045

**Published:** 2020-10-01

**Authors:** Gervason Moriasi, Anthony Ireri, Mathew Ngugi

**Affiliations:** ^1^Kenyatta University, Department of Biochemistry Microbiology and Biotechnology, P.O. Box 43844-00100, Nairobi, Kenya; ^2^Mount Kenya University, Department of Medical Biochemistry, P.O. Box 342-01000, Thika, Kenya; ^3^Kenyatta University, Department of Educational Psychology, P.O. Box 43844-00100, Nairobi, Kenya

## Abstract

Over 50 million persons are living with cognitive deficits worldwide, with over 80% of these individuals living in the developing world. The number of affected persons is projected to go over 152 million by the year 2050. Current drugs used for cognitive impairment are debatably ineffective, costly, inaccessible, and associated with undesirable events that call for the search for alternative and complementary approaches. Plants are arguably affordable, accessible, and efficacious. However, despite the reported healing claims, scientific data validating these claims are lacking. *L. eriocalyx* is traditionally used for the management of various conditions, including cognitive impairment but has not been scientifically explored. In this study, the Morris Water Maze (MWM) method was used to evaluate *in vivo* cognitive-enhancing effects of studied extracts of *L. eriocalyx.* Furthermore, following MWM experiments, brains were dissected and processed, and malondialdehyde profiles were determined. Qualitative phytochemical profiles of the studied plant extracts were also determined. The results showed that mice that were treated with the studied plant extracts took significantly shorter transfer latencies, navigation distances, and significantly longer latencies in the target quadrant (NW) (*p* < 0.05) compared with the negative control mice, indicating cognitive-enhancing activities. Furthermore, cognitively impaired mice that received the studied plant extracts had significantly lower MDA profiles compared with the MDA profile of the negative control group mice (*p* < 0.05). The cognitive-enhancing and MDA profile lowering effects were attributed to the presence of antioxidant phytoconstituents that ought to have modulated the redox state, thereby attenuating brain damage. These extracts can be, therefore, used for the management of cognitive deficits. Further studies leading to isolation and characterization of active molecules for cognitive impairment are recommended. Furthermore, the precise mechanism(s) through which these extracts exert their pharmacologic activity should be established.

## 1. Introduction

According to the Alzheimer's Association [1], cognitive impairment poses a physical, emotional, and financial burden to the affected patients and their caregivers. Globally, the population of people living with cognitive impairment is estimated to be 50 million. Furthermore, these figures are feared to escalate to 88 million by the year 2030 and 152 million by the year 2050. Moreover, over 80% of cognitively impaired persons are expected to be living in low- and middle-income countries, which poses a substantial economic burden [2, 3].

Various neuromodulators and neurotransmitters, including acetylcholine, nitric oxide, *γ*-aminobutyric acid (GABA), and endogenous antioxidants, among others, play essential roles in the central cholinergic system [4]. The dysfunction of the central cholinergic system causes cognitive deficits witnessed in neurodegenerative and associated disorders. Cognitive impairment is attributable to the deficits in brain functions, including learning, memory, attention, among others, which acetylcholine neurotransmitter modulate in the amygdala, hippocampus, and striatal regions of the brain [5–7]. Therefore, any disorder affecting these brain regions or the activity of acetylcholine impairs these cognitive functions.

Oxidative damage to the brain has been implicated as the primary cause of brain dysfunction and cognitive impairment [8, 9]. Research has shown that the high metabolic activity, higher concentration of polyunsaturated fatty acids (PUFA), and mitochondrial abnormalities all contribute to oxidative damage [8–10]. The high concentrations of PUFA make the brain more vulnerable to oxidative attack, manifesting in high concentrations of malondialdehyde (MDA), the major reactive oxygen metabolite (ROM), which further add up to the brain's detriment [11].

Attempts to curb cognitive deficits by the use of psychopharmacologic agents have been made [12–16]. However, the low efficacy and associated undesirable events caused by drugs used in conventional healthcare for cognitive impairment have rendered them ineffective [17]. For instance, Rivastigmine causes severe diarrhea, nausea, dizziness, and headache. Besides, some of these medications, like galantamine, have proved to be ineffective in ameliorating cognitive impairment [1, 17, 18]. Additionally, all the conventional medicines used against cognitive impairment only manage symptoms without curing the underlying cause [18].

Due to the rising statistics of affected subjects and the inefficiency of treatments, the need for alternative mechanisms and agents is imperious. Plants offer a feasible alternative as they are arguably safe, affordable, and accessible, and they contain a myriad of bioactive compounds that exert pharmacologic activity through multitarget sites leading to increased potency, thus ameliorating dementia [19, 20]. Phenolic compounds have the broadest spectrum of bioactivity, mainly due to their redox homeostasis restoring ability [21]. In the Kenyan Traditional Medicine practice, *L. eriocalyx* is used for enhancing cognition, management of diabetes mellitus, and high blood pressure, among other cognitive deficit-associated conditions [22]. Previous studies have demonstrated the antiplasmodial and analgesic properties of the root bark and leaf extracts of *L. eriocalyx* [23, 24]. However, this plant has not been explored for its cognitive-enhancing activities.

## 2. Materials and Methods

### 2.1. Plant Collection and Processing

Fresh stem barks of *L. eriocalyx* were collected from Cianyi village, Muchomoke sublocation, Gitiburi location, Siakago division, Mbeere North subcounty in Embu County, Kenya, in the plant's natural habitat. They were transported to Kenyatta University, in the Department of Biochemistry, Microbiology, and Biotechnology laboratories, where they were chopped off into small pieces, evenly spread on the bench, and allowed to naturally dry at room temperature for 14 days. Regular grabbling was done to ensure uniform drying and deter moisture trapping. During plant collection, a voucher specimen was prepared and identified with the help of a taxonomist at the Department of Plant Sciences of Kenyatta University where voucher number GM002/2017 was assigned, and the specimen deposited for future reference. After drying, the barks were ground into a coarse powder with the help of a plant mill and kept in a khaki envelope, which was sealed and kept on a clean, dry shelf awaiting extraction.

### 2.2. Experimental Animals

Swiss albino mice (4-5 weeks, 24 ± 2 g bw) were sourced from the Kenya Medical Research Institute (KEMRI), Nairobi, kept in standard laboratory conditions, and housed in polypropylene rectangular cages measuring 30 cm × 20 cm × 13 cm with softwood shavings as bedding material. They were offered standard rodent pellets and clean water *ad-libitum* and kept at a natural 12-hour day-night cycle. Before experimentation, they were allowed 72 hours for habituation. Humane handling and standard protocols guiding laboratory animal manipulation, care, and disposal outlined by the National Research Council [25] were followed throughout the experimentation period. The National Commission for Science, Technology, and Innovation (NACOSTI) authorized this study and granted ethical approval under license number NACOSTI/P/19/2080.

### 2.3. Extraction Methods

Extraction was performed according to the method described by Harborne [26]. Briefly, the methanolic extract was obtained by cold maceration of 200 g of *L. eriocalyx* bark powder in 750 ml of methanol (AR grade) in a 1 liter conical flask and regularly agitated for 48 hours. The mixture was carefully decanted and filtered using Whatman filter papers(No. 1) and concentrated *in vacuo* with a rotary evaporator at 50°C. The extract was transferred into a clean, dry, labelled, preweighed universal glass bottle and kept in a hot-air oven set at 35°C for five days to allow for complete drying.

On the other hand, the aqueous extract was obtained by warming 50 g of the powdered *L. eriocalyx* bark powder at 58°C in distilled water on a water bath for two hours. The resulting mixture was cooled to room temperature, filtered through a Whatman filter paper (No. 1), and transferred into clean freeze-drying flasks. The flasks were covered with solid CO_2_-acetone mixture and then fitted into a freeze dryer for lyophilization for 48 hours. The dry and lyophilized extracts were transferred into clean, dry, preweighed, and labeled universal glass bottles. The percentage yields of the individual extracts were determined using the formula described by Harborne [26] and modified by Truong et al. [27]:

(1) $$\begin{array}{c} \%\text{ yield}=\frac{\text{weight of extract}}{\text{weight of macerated powder}}\times 100. \end{array}$$

### 2.4. Investigation of *In Vivo* Cognitive-Enhancing Effects of the Aqueous and Methanolic Stem Bark Extracts of *L. eriocalyx*

The Morris water maze (MWM) method described by Morris [28, 29] was used in this study for the determination of *in vivo* cognitive-enhancing effects of the aqueous and methanolic stem bark extracts of *L. eriocalyx* in mice models.

#### 2.4.1. Setup

The maze consisted of a white circular tank (110 cm in diameter and 45 cm in height) with a featureless inner surface. It was filled with clean water, in which 750 g of powdered milk was mixed, to a height of 30 cm, to form an opaque pool, and its temperature was maintained at 26 ± 1°C. A white escape platform (10 cm diameter and 29 cm in height) was placed in the center of the northwest (NW) quadrant of the pool and submerged 1 cm below the water surface to make it invisible at the water level. On the walls of the maze, manila papers of blue, green, pink, and yellow colors were mounted to theWest (W), North (N), South (S), and East (E) quadrants, respectively, as visual cues before introducing the mice. The continuous location of each swimming mouse, from the start position to the top of the platform, was monitored with the help of a digital Sony video camera that was mounted 1.5 meters above the maze [28–30].

#### 2.4.2. Training

Before the experiment, each mouse was trained four times to swim for 60 seconds in the presence and absence of a visible escape platform with an intertraining break of 20 minutes.

#### 2.4.3. Acquisition

In the acquisition period, the water level in the maze was adjusted to 1 cm above the escape platform, which was centered in the northwest (NW) quadrant to make it invisible at the water level. Mice were subjected to three sessions each day for three consecutive days with an intertrial break of 20 minutes. The starting point was predetermined (southeast, SE) and remained unchanged for the entire experimental period. A digital video recorder was used to record transfer latency and navigation distance for each mouse. The recorded video clips were fed into Any-Maze software (version 6.05) for quantitative data acquisition.

#### 2.4.4. Probe Trial

The experimental mice were introduced into the maze without the escape platform on the fourth day (Day 4) for a single probe trial to assess their learning and spatial memory retention. During this session, the time spent in the target quadrant (NW, the correct location of the escape platform) was recorded (NW quadrant latency).

#### 2.4.5. Experimental Design

In this study, a completely controlled, randomized study design was adopted from which an experimental design was derived. For both the aqueous and methanolic stem bark extracts of *L. eriocalyx*, thirty Swiss albino mice were used. Five experimental mice were randomly selected and allotted into six groups (I, II, III, IV, V, and VI).

Group I (normal control) mice were orally administered with normal saline at a dose of 10 ml/kg bw. On the other hand, Group II (negative control) mice received normal saline (10 ml/kg bw) and scopolamine (1 mg/kg bw), whereas, Group III (positive control) were orally administered with Donepezil at a dose level of 1 mg/kg bw and scopolamine (1 mg/kg bw) intraperitoneally. Besides, Groups IV, V, and VI (experimental) received either the aqueous or the methanolic stem bark extracts of *L. eriocalyx* at dose levels of 50 mg/kg bw, 100 mg/kg bw, and 200 mg/kg bw, respectively, orally and scopolamine (1 mg/kg bw) intraperitoneally. In all treatment groups, scopolamine was administered after 30 minutes following oral administration of respective treatments.

### 2.5. *Ex Vivo* Determination of Malondialdehyde (MDA) Profiles in the Brains of Scopolamine-Induced Cognitively Impaired Mice

The Thiobarbituric Acid Reactive Substances (TBARS) assay technique was used to determine the *ex vivo* effects of the aqueous, and methanolic stem bark extracts of *L. eriocalyx* on MDA concentration, a marker of lipid peroxidation, in the brains of cognitively impaired mice were adopted [31, 32]. At the end of the MWM experiment, all the mice were sacrificed by cervical dislocation, after which the whole brains were quickly dissected under standard conditions and eviscerated with ice-cold saline before storage at −20°C.

During the assay, the brain samples were retrieved from the freezer and thawed, and each brain sample was homogenized in 10 ml of ice-cold phosphate buffer (0.1 M: pH 7.4). The reaction mixtures comprised of 1.5 ml of 0.8% thiobarbituric acid, 1.5 ml of 20% acetic acid (pH 3.5), 0.2 ml of 8.1% sodium dodecyl sulphate, and 0.1 ml of the brain tissue homogenates. The reaction mixtures were incubated in a boiling water bath (100°C) for 1 hour and then cooled to room temperature (25°C). After that, 5 ml of *n*-butanol/pyridine (15 : 1) mixture and 1 ml of distilled water were added to the mixtures, and vigorously vortexed before they were centrifuged (2,500 rpm; 20 minutes). Following centrifugation, the supernatants were separated and their absorbances measured at 532 nm using a dual-beam UV-Vis spectrophotometer (Shimadzu UV-Vis 1600). The obtained absorbance values were used to determine the amount of MDA (*μ*mol/g of tissue) using a molar extinction coefficient (*ε*) of 1.56 × 10^5^ M^−1^cm^−1^ [31, 32].

### 2.6. Qualitative Phytochemical Screening

Qualitative tests for various phytochemical compounds in the aqueous and methanolic stem bark extract of *L. eriocalyx* were also done in this study. The standard phytochemical screening methods described by Harborne [26] and modified by Bello et al. [33], Jared et al. [34], Moriasi et al. [35], and Kumar et al., [36] were followed.

## 3. Data Management and Statistical Analysis

The yields of the studied plant extracts were expressed as a percentage of weights of powders macerated. Quantitative data for the MWM test were obtained from the Any-Maze tracking software version 6.05. The MWM and MDA profile data were tabulated on Excel spreadsheets (Microsoft Office 365), and exported to Minitab version 19.1 statistical software (State College, Pennsylvania) for analysis. In the Minitab platform, descriptive statistics were performed, and resultant values were expressed as $\bar{x}$ ± SEM.

Also, One-Way ANOVA was performed to determine statistical significance among the normal control, positive control, negative control, and the experimental groups at *α*_0.05_ followed by Fisher's LSD *post hoc* test for pairwise comparisons and separation of means. An unpaired Student's *t* test statistic was done to compare the effects of the two independent extracts (aqueous and methanolic) on the investigated parameters, at each of the studied dose levels, at a 95% confidence level. In all instances, *p* < 0.05 was considered statistically significant. Graphs and tables were used to present quantitative data, while qualitative data from phytochemical screening were presented in a table.

## 4. Results

### 4.1. Percentage Yield

Following extraction, the percentage yields of the studied plant extracts were determined. In general, the yield of the aqueous stem bark extract of *L. eriocalyx* was higher than the yield of the methanolic extract. The results are presented in Table 1.

### 4.2. *In Vivo* Cognitive-Enhancing Effects of the Aqueous and Methanolic Stem Bark Extracts of *L. eriocalyx*

To appraise the cognitive-enhancing effects of the studied plant extracts in experimental mice that were subjected to the MWM task, the transfer latency, navigation distance, and latency time in the target quadrant (NW) were measured.

#### 4.2.1. Effects of the Aqueous and Methanolic Stem Bark Extracts of *L. eriocalyx* on Transfer Latency Taken by Experimental Mice during Spatial Learning Acquisition Training

**Figure 1:**
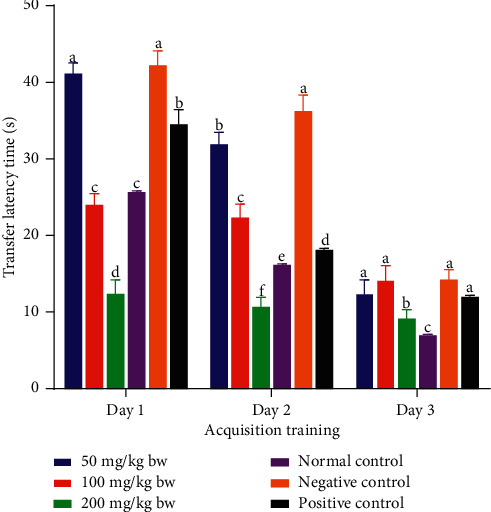
Transfer latencies taken by experimental mice treated with the aqueous stem bark extract of *L. eriocalyx* during the acquisition training period. Bars with the same letter within the same acquisition training day are not significantly different (*p* > 0.05; one-way ANOVA followed by Fisher's LSD test).

The results showed that, on acquisition training day 1, the transfer latency taken by mice that were treated with the aqueous stem bark extract of *L. eriocalyx*, at a dose level of 50 mg/kg bw, was not significantly different from the transfer latency taken by mice in the negative control group (*p* > 0.05; Figure 1). Similarly, the transfer latency taken by the mice that received the aqueous stem bark extract of *L. eriocalyx* at 100 mg/kg bw dose level was not significantly different from the time taken by mice in the normal control group in this particular day (*p* > 0.05; Figure 1). Notably, the experimental mice which were administered with the aqueous stem bark extract of *L. eriocalyx* at a dose level of 200 mg/kg bw took the least transfer latency to complete the MWM task compared with the latencies taken by mice in all the other groups on this day (*p* < 0.05; Figure 1).

On the second day of acquisition training, we noted significant differences in transfer latencies that were taken by mice to complete the MWM task (*p* < 0.05). The obtained results revealed that the experimental mice that were administered with the aqueous stem bark extract of *L. eriocalyx*, at all the studied dose levels, took significantly shorter transfer latencies than the time taken by mice in the negative control group (*p* < 0.05; Figure 1). Notably, the mice that received this extract, at a dose of 200 mg/kg bw, took the least transfer latency, which compared significantly with the times taken by the mice in all the other groups (*p* < 0.05; Figure 1).

On the third day, no significant differences in transfer latencies taken by mice in the positive control group and negative control group and those that were treated with the aqueous stem bark extract of *L. eriocalyx*, at doses of 50 mg/kg bw and 100 mg/kg bw were observed (*p* > 0.05; Figure 1). However, the mice that were administered with this extract, at a dose of 200 mg/kg bw, took significantly shorter transfer latency to complete the MWM task compared with the times takes taken mice in both the negative and positive control groups (*p* < 0.05; Figure 1).

**Figure 2:**
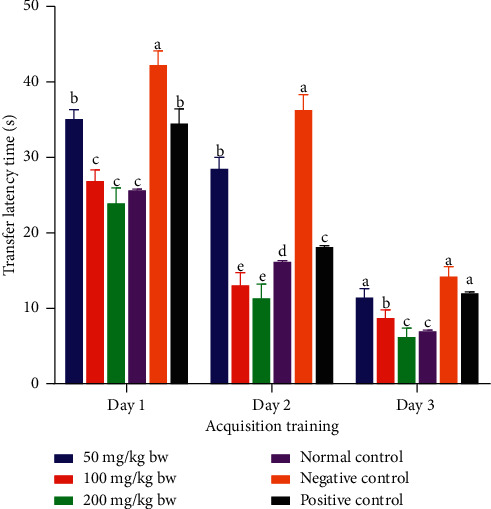
Transfer latencies taken by experimental mice treated with the methanolic stem bark extract of *L. eriocalyx* during the acquisition training period. Bars with the same letter within the same acquisition training day are not significantly different (*p* > 0.05; one-way ANOVA followed by Fisher's LSD test).

On the other hand, during acquisition training day 1, the experimental mice that were administered with the methanolic stem bark extract of *L. eriocalyx* at a dose of 50 mg/Kg bw took a comparably similar transfer latency to the latency of mice in the positive control group (*p* > 0.05; Figure 2). Furthermore, the transfer latencies taken by mice which received 100 mg/kg bw and 200 mg/kg bw of the methanolic stem bark extract of *L. eriocalyx*, were not significantly different from the transfer latency taken by mice in the normal control group on the same day (*p* > 0.05; Figure 2). Conversely, on the same day, the transfer latency taken by mice in the negative control group was significantly longer than the transfer latencies taken by mice in all the other groups (*p* < 0.05; Figure 2).

Moreover, on the second day of acquisition training, the mice which were administered with the methanolic stem bark extract of *L. eriocalyx*, at all the three studied dose levels, took significantly shorter transfer latencies compared with the time taken by mice in the negative control group (*p* < 0.05; Figure 2). Notably, the mice that received this extract, at doses of 100 mg/kg bw and 200 mg/kg bw, took significantly shorter transfer latencies compared with those of mice in all the other groups (*p* < 0.05; Figure 2).

On the third day of acquisition training, there were no significant differences in transfer latencies taken by mice that received the methanolic stem bark extract of *L. eriocalyx*, at a dose of 50 mg/kg bw, and of those in the negative and positive control groups (*p* > 0.05; Figure 2). However, significantly shorter transfer latencies were observed in mice that were administered with 100 mg/kg bw and 200 mg/kg bw of this extract, on this acquisition training day, compared with the times taken by mice in the negative and positive control groups (*p* < 0.05; Figure 2). Notably, the transfer latency taken by mice, which were treated with 200 mg/Kg bw, was not significantly different from that of mice in the positive control group (*p* > 0.05; Figure 2).

**Figure 3:**
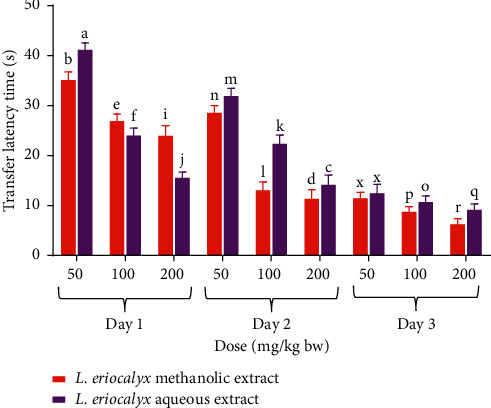
Comparison between the effects of the studied plant extracts on transfer latencies that were taken by mice during the acquisition training period. Bars with the same letter within the same dose level and acquisition training day are not significantly different (*p* > 0.05; unpaired Student's *t* test).

Furthermore, a comparison between the effects of the studied plant extracts on experimental mice's transfer latencies during the acquisition training days (Days 1–3) was done in this study. The results revealed that, on the first day of acquisition training, the mice that received 50 mg/kg bw of the methanolic stem bark extract of *L. eriocalyx*, took significantly shorter time compared with the time taken by those treated with a similar dose of the aqueous extract, to complete the MWM task (*p* < 0.05; Figure 3). However, the mice that received the aqueous stem bark extract of *L. eriocalyx*, at dose levels of 100 mg/kg bw and 200 mg/kg bw, took significantly shorter latencies than the latencies taken by mice that received similar doses of the methanolic stem bark extract (*p* < 0.05; Figure 3).

On the other hand, on the second day of acquisition training, the transfer latencies taken by mice which received the methanolic stem bark extract of *L. eriocalyx*, at all dose levels, were significantly shorter than those taken by mice which were treated with the aqueous stem bark extract of the same plant at similar doses (*p* < 0.05; Figure 3).

Besides, on the third acquisition training day, the latencies taken by mice that were administered with the studied plant extracts, at a dose of 50 mg/kg bw, were not significantly different (*p* > 0.05; Figure 3). However, at dose levels of 100 mg/kg bw and 200 mg/kg bw, the latencies taken by mice that received the methanolic stem bark extract of the studied plant were significantly shorter than those taken by mice treated with the aqueous extract (*p* < 0.05; Figure 3).

#### 4.2.2. Effects of the Aqueous and Methanolic Stem Bark Extracts of *L. eriocalyx* on Transfer Latencies Taken by Mice following Scopolamine-Induced Cognitive Impairment

**Figure 4:**
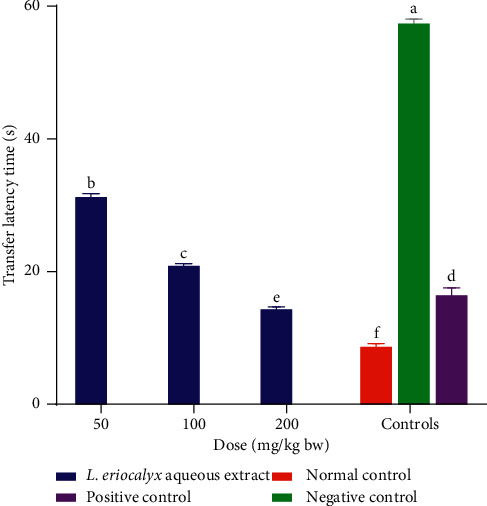
Effect of the aqueous stem bark extract of *L. eriocalyx* on transfer latencies taken by scopolamine-induced cognitively impaired mice. Bars with the same letter are not significantly different (*p* > 0.05; one-way ANOVA followed by Fisher's LSD test).

Upon administration of the aqueous stem bark extract of *L. eriocalyx* into cognitively impaired experimental mice, at different doses, there were remarkable reductions in transfer latencies (*p* < 0.05; Figure 4). The cognitive-impaired mice that were treated with the aqueous stem bark extract of *L. eriocalyx*, at dose levels of 50 mg/kg bw and 100 mg/kg bw, took significantly longer transfer latency, compared with that taken by mice in the normal control group (*p* < 0.05). It was, however, noted that, at the extract dose level of 200 mg/kg bw, the transfer latency taken by the experimental mice was significantly shorter compared with the transfer latency taken by the mice in the positive control group (*p* < 0.05; Figure 4).

**Figure 5:**
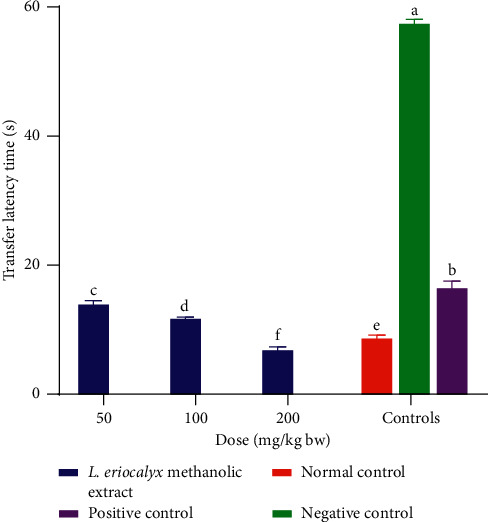
Effect of the methanolic stem bark extract of *L. eriocalyx* on the transfer latencies taken by scopolamine-induced cognitively impaired mice. Bars with the same letter are not significantly different (*p* > 0.05; one-way ANOVA followed by Fisher's LSD test).

Besides, the transfer latencies taken by mice, which were treated with the methanolic stem bark extract of *L. eriocalyx* at all the three studied dose levels, were significantly shorter compared with latencies taken by mice in the positive and negative control groups (*p* < 0.05; Figure 5). Remarkably, the mice that were treated with the methanolic extract, at a dose of 200 mg/kg bw, recorded a significantly shorter transfer latency compared with the transfer latencies taken by the mice in all the control groups (*p* < 0.05; Figure 5).

**Figure 6:**
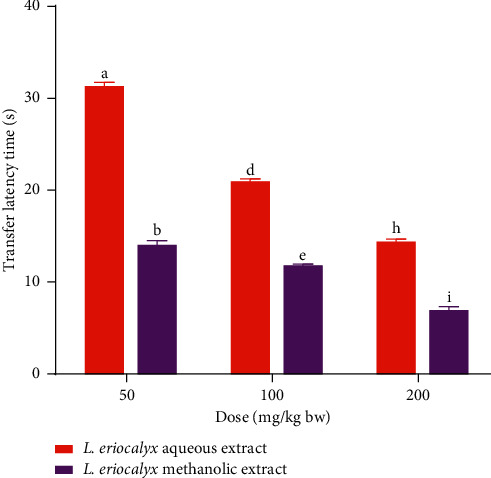
Comparison between transfer latencies taken by scopolamine-induced cognitive-impaired mice treated with the aqueous and methanolic stem bark extract of *L. eriocalyx*. Bars with the same letter within the same dose level are not significantly different (*p* > 0.05; unpaired Student's *t* test).

Moreover, a comparison between the effects of the aqueous and methanolic stem bark extracts of *L. eriocalyx*, at the studied doses, on transfer latencies taken by scopolamine-induced cognitively impaired mice was done. The results revealed that, at all the three tested doses, the transfer latencies taken by mice that received the methanolic stem bark extract of *L. eriocalyx* were significantly shorter than those of mice that were administered with the aqueous extract (*p* < 0.05; Figure 6).

#### 4.2.3. Effects of the Aqueous and Methanolic Stem Bark Extracts of *L. eriocalyx* on Navigation Distance Covered by Experimental Mice during Spatial Learning Acquisition Training

**Figure 7:**
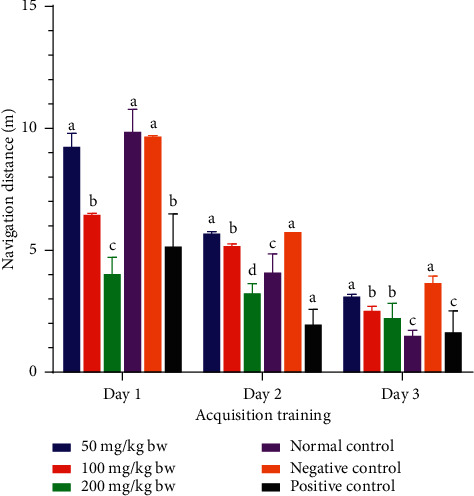
Effects of the aqueous stem bark extract of *L. eriocalyx* on navigation distances covered by experimental during the acquisition training period. Bars with the same letter within the same acquisition training day are not significantly different (*p* > 0.05; one-way ANOVA followed by Fisher's LSD test).

On the first acquisition training day, the mice which received the aqueous stem bark extract of *L. eriocalyx* at a dose of 50 mg/kg bw covered a comparatively similar navigation distance to that covered by mice in the negative and normal control groups (*p* > 0.05; Figure 7). Also, the navigation distance covered by mice that were treated with the aqueous extract, at a dose of 100 mg/kg bw, was not significantly different from the navigation distance covered by mice in the positive control group (*p* > 0.05; Figure 7). Remarkably, the mice that were administered with the aqueous stem bark extract of *L. eriocalyx*, at a dose of 200 mg/kg bw, covered a significantly shorter distance compared with the distances covered by mice in all the other groups (*p* < 0.05; Figure 7).

On the second acquisition training day, no significant differences in navigation distances covered by mice treated with 50 mg/kg bw of the aqueous stem bark extract of *L. eriocalyx* and those in the negative control group (*p* > 0.05; Figure 7). However, the navigation distance covered by mice that received 100 mg/kg bw of this extract was significantly shorter than the distance covered by the mice in the negative control group (*p* < 0.05; Figure 7). Additionally, the navigation distance covered by mice that were treated with the aqueous stem bark extract of *L. eriocalyx* at a dose of 200 mg/kg bw was significantly shorter than the distance covered by mice in the normal and negative control groups (*p* < 0.05; Figure 7). Also, the mice in the positive control group covered a significantly shorter distance in this acquisition training day compared with the distances covered by mice in all the other groups (*p* < 0.05; Figure 7).

The results further revealed that, on the third day of acquisition training, the navigation distance covered by mice treated with 50 mg/kg bw of the aqueous stem bark extract of *L. eriocalyx* was not significantly different from the navigation distance covered by mice in the negative control group (*p* > 0.05; Figure 7). Besides, there was no significant difference in navigation distances covered by mice that were treated with 100 mg/kg bw and 200 mg/kg bw doses of this extract on this day (*p* > 0.05; Figure 7). However, the mice in the normal and positive control groups covered the shortest navigation distances, which compared significantly with the distances covered by mice in all the other groups (*p* > 0.05; Figure 7).

**Figure 8:**
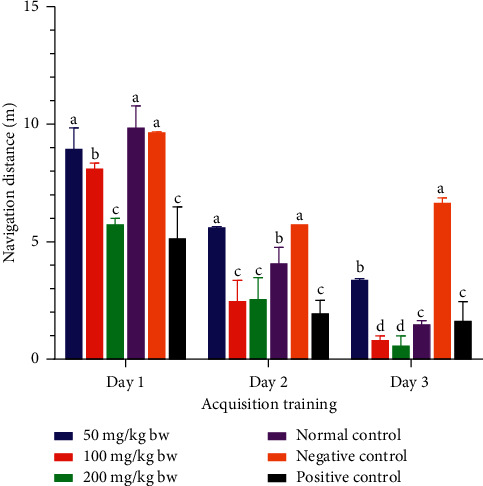
Effect of the methanolic stem bark extract of *L. eriocalyx* on the navigation distances covered by experimental mice during the acquisition training period. Bars with the same letter within the same acquisition training day are not significantly different (*p* > 0.05; one-way ANOVA followed by Fisher's LSD test).

On the other hand, the mice that were treated with the methanolic stem bark extract of *L. eriocalyx* at a dose of 50 mg/kg bw navigated a comparably similar distance to the distance covered by mice in the normal and negative control groups on the first day of acquisition training (*p* > 0.05; Figure 8). However, on the same day (acquisition training day 1), the mice that were administered with the methanolic stem bark extract of *L. eriocalyx* at 100 mg/kg bw and 200 mg/kg bw dose levels covered significantly shorter navigation distances than the distances navigated by mice in the normal and negative control groups (*p* < 0.05; Figure 8). Notably, the distance navigated by mice that received a 200 mg/kg bw dose of the methanolic stem bark extract of *L. eriocalyx* was not significantly different from the navigation distance covered by mice in the positive control group (*p* > 0.05; Figure 8).

On the second acquisition training day, there was no significant difference in navigation distance covered by mice that received a 50 mg/kg bw dose of the methanolic stem bark extract of *L. eriocalyx* compared with the distance which was covered by mice in the negative control group (*p* > 0.05; Figure 8). However, on this day, the navigation distances covered by mice that were administered with 100 mg/kg bw and 200 mg/kg bw of the methanolic stem bark extract of *L. eriocalyx* were significantly shorter than the distances covered by mice in both the normal and negative control groups (*p* < 0.05; Figure 8) and comparably similar to the distance covered by the positive control group mice (*p* > 0.05; Figure 8).

On acquisition day 3, the results revealed that the mice which received the methanolic stem bark extract of *L. eriocalyx,* at all the three studied dose levels, navigated significantly shorter distances than the distance navigated by the negative control group mice (*p* < 0.05; Figure 8). Remarkably, the mice into which 100 mg/kg bw and 200 mg/kg bw doses of this extract were administered covered significantly shorter navigation distances compared with the distances navigated by mice that received a 50 mg/kg bw dose of this extract and those in all the control groups (*p* < 0.05; Figure 8). There was no significant difference between the navigation distance covered by the normal control mice compared with the distance covered by the positive control group mice in this day (*p* > 0.05; Figure 8).

Furthermore, a comparison between the effects of the aqueous and methanolic stem bark extracts of *L. eriocalyx*, at the studied doses, on navigation distances covered by experimental mice during spatial learning acquisition training was performed.

**Figure 9:**
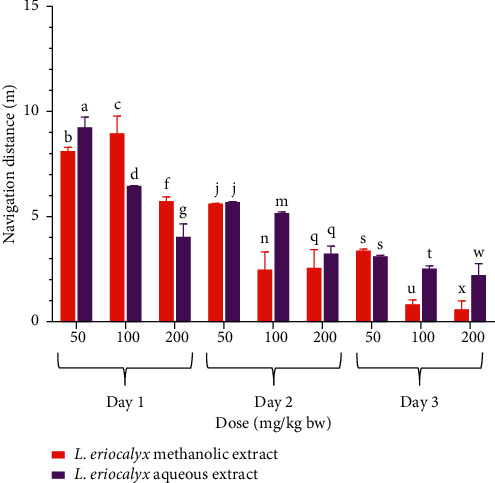
Comparison between the navigation distances that were covered by which received the aqueous and methanolic stem bark extracts of *L. eriocalyx* during the acquisition training period. Bars with the same letter within the same dose level and training day are not significantly different (*p* > 0.05; unpaired Student's *t* test).

The results showed, during acquisition training day 1, the mice that were treated with the aqueous stem bark extract of *L. eriocalyx*, at a dose of 50 mg/kg bw, covered a significantly longer distance than the distance covered by mice which received a similar dose of methanolic stem bark extract (*p* < 0.05; Figure 9). However, during the same acquisition training day, the navigation distances covered by mice which received the methanolic extract of the studied plant, at doses of 100 mg/kg bw and 200 mg/kg bw, were significantly longer than the distances covered by their counterparts that received the aqueous extract, at similar dose levels (*p* < 0.05; Figure 9).

On the other hand, during the second acquisition training day, there was no significant difference in navigation distances recorded between the experimental mice which were administered with the studied plant extracts, at dose levels of 50 mg/kg bw and 200 mg/kg bw (*p* > 0.05; Figure 9). However, the mice which received 100 mg/kg bw of the methanolic stem bark extract of the studied plant covered a significantly shorter distance compared with that covered by those that received a similar dose of the aqueous stem bark extract (*p* < 0.05; Figure 9).

Besides, on the third acquisition training day, no significant difference between navigation distances was observed in mice, which received 50 mg/kg bw of the studied plant extracts (*p* > 0.05). However, during the same day, the mice which received the methanolic extract, at dose levels of 100 mg/kg bw and 200 mg/kg bw, of the methanolic extract covered significantly shorter distances than those covered by mice which were treated with the aqueous extract, at the same dose levels (*p* < 0.05; Figure 9).

#### 4.2.4. Effects of the Aqueous and Methanolic Stem Bark Extracts of *L. eriocalyx* on the Navigation Distance Covered by Mice following Scopolamine-Induced Cognitive Impairment

**Figure 10:**
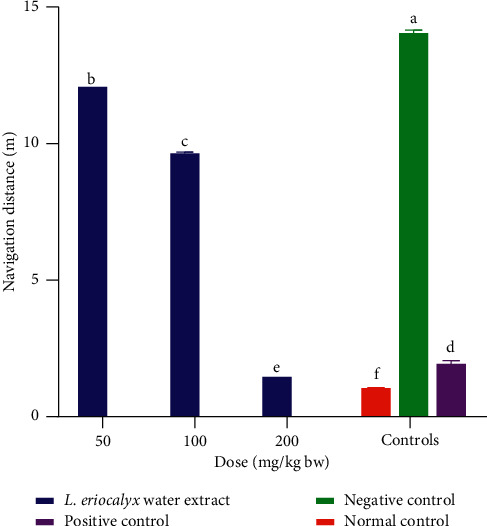
Effects of the aqueous stem bark extracts of *L. eriocalyx* on the navigation distance covered by scopolamine-induced cognitively impaired mice. Bars with the same letter are not significantly different (*p* > 0.05; one-way ANOVA followed by Fisher's LSD test).

The results showed that the mice which were administered with the aqueous stem bark extract of *L. eriocalyx*, at a dose of 50 mg/kg bw, covered a significantly longer distance to reach the escape platform compared with the distance covered by the mice in the normal and positive control groups (*p* < 0.05; Figure 10). A similar trend was depicted by the mice that were treated with the aqueous stem bark extract of *L. eriocalyx*, at a dose of 100 mg/kg bw (*p* < 0.05; Figure 10). However, at a dose level of 200 mg/kg bw, a significantly shorter navigation distance was covered by the experimental mice treated with the aqueous stem bark extract of the studied plant compared with the distance covered by the mice in the positive and negative control groups (*p* < 0.05; Figure 10).

**Figure 11:**
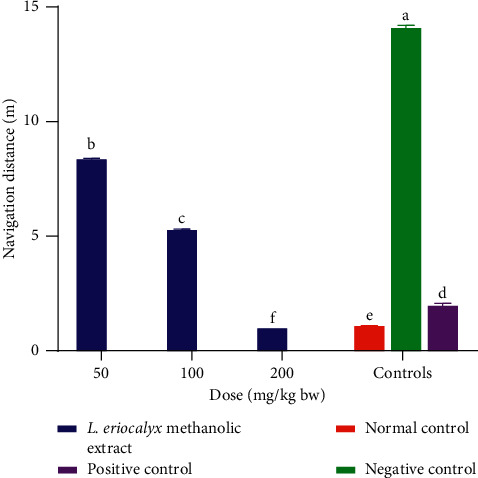
Effects of the methanolic stem bark extracts of *L. eriocalyx* on the navigation distance covered by scopolamine-induced cognitively impaired mice. Bars with the same letter are not significantly different (*p* > 0.05; one-way ANOVA followed by Fisher's LSD test).

On the other hand, orally administered methanolic stem bark extracts of *L. eriocalyx*, at a dose of 100 mg/kg bw, in experimental mice significantly reduced navigation distance compared with the distance covered by the mice that received the extract at a dose of 50 mg/kg bw (*p* < 0.05; Figure 11). Also, the mice that received the methanolic stem bark extract of *L. eriocalyx*, at a dose of 200 mg/kg bw, covered significantly shorter navigation distances compared with the distances covered by the mice in all control groups (*p* < 0.05; Figure 11).

**Figure 12:**
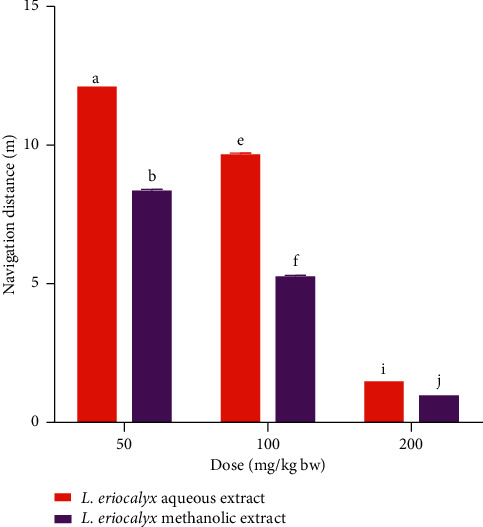
Comparison between the effects of the aqueous and methanolic stem bark extracts of *L. eriocalyx* on the navigation distances covered by scopolamine-induced cognitively impaired mice. Bars with the same letter within the same dose level are not significantly different (*p* > 0.05; unpaired Student's *t* test).

Additionally, a comparison between the effects of the aqueous and methanolic stem bark extracts of *L. eriocalyx* on navigation distance of experimental mice following scopolamine-induced cognitive impairment was done. The results showed that, at all the studied dose levels, the mice which were orally administered with the methanolic stem bark extract of *L. eriocalyx* covered significantly shorter navigation distances than those covered by mice which received the aqueous extract of this plant, at similar dose levels (*p* < 0.05; Figure 12).

#### 4.2.5. Effects of the Aqueous and Methanolic Stem Bark Extracts of *L. eriocalyx* on Latency Taken by Scopolamine-Induced Cognitively Impaired Mice in the NW Quadrant

**Figure 13:**
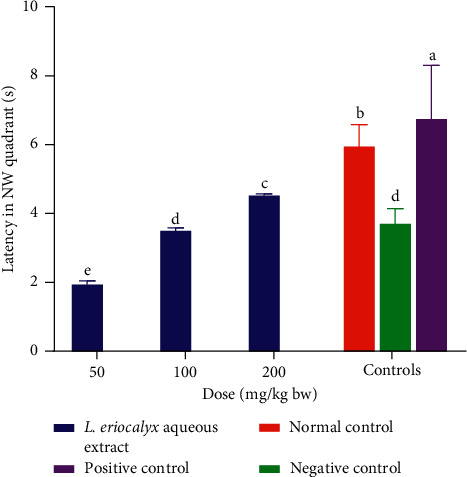
Effects of the aqueous stem bark extract of *L. eriocalyx* on latency taken by scopolamine-induced cognitively impaired mice in the NW quadrant. Bars with the same letter are not significantly different (*p* > 0.05; one-way ANOVA followed by Fisher's LSD test).

The results showed that the cognitively impaired experimental mice that were treated with the aqueous stem bark extract of *L. eriocalyx*, at all the three studied doses, spent significantly shorter latencies in the NW quadrant compared with the times spent by both the normal and positive control mice in the same quadrant (*p* < 0.05; Figure 13). However, no significant differences in the times spent in the NW quadrant by mice in the negative control group and those that were treated with the aqueous stem bark extract of *L. eriocalyx* at a dose level of 100 mg/kg bw (*p* > 0.05; Figure 13).

Generally, a dose-dependent increase in latency time in the NW quadrant was observed in cognitively impaired mice, which were treated with the aqueous stem bark extract of *L. eriocalyx* across the three studied dose levels (Figure 13).

**Figure 14:**
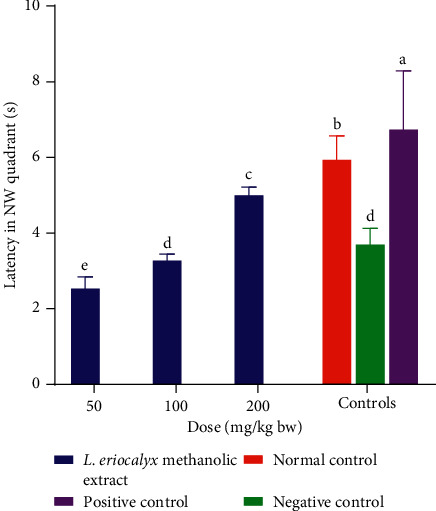
Effects of the methanolic stem bark extracts of *L. eriocalyx* on latency taken by scopolamine-induced cognitively impaired mice in the NW quadrant. Bars with the same letter are not significantly different (*p* > 0.05; One-Way ANOVA followed by Fisher's LSD).

On the other hand, the cognitively impaired mice that received the methanolic stem bark extract of *L. eriocalyx*, at a dose of 50 mg/kg bw, spent significantly shorter latency in the NW quadrant compared with the latencies taken by mice in the rest of the groups in the same quadrant by (*p* < 0.05; Figure 14). However, the latency taken by mice that were administered with methanolic stem bark extract of *L. eriocalyx*, at a dose of 100 mg/kg bw, in the NW quadrant was not significantly different from the latency of mice in the negative control in this quadrant (*p* > 0.05; Figure 14). Besides, the latency of mice treated with 200 mg/Kg bw of the studied plant in the NW quadrant was significantly longer than that taken by the negative control mice (p<0.05; Figure 14).

Furthermore, the times spent in the NW quadrant by mice in the normal and positive control groups were significantly longer than the times spent by the mice in the rest of the groups in the same quadrant (*p* < 0.05; Figure 14).

**Figure 15:**
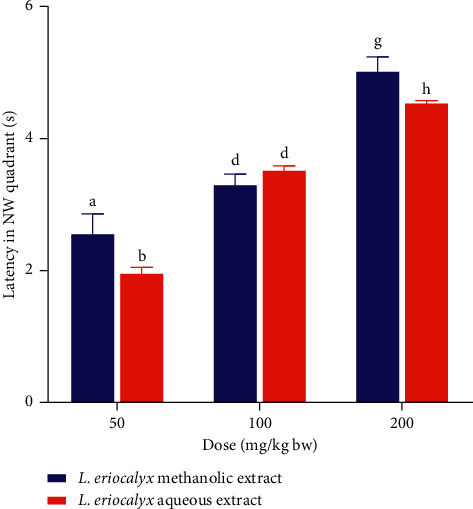
Comparison between the effects of the aqueous and methanolic stem bark extracts of *L. eriocalyx* on latency taken by scopolamine-induced cognitively impaired mice in the NW quadrant. Bars with the same letter within the same dose level are not significantly different (*p* > 0.05; unpaired Student's *t* test).

In this study, a comparison between the effects of the aqueous and methanolic stem bark extracts of *L. eriocalyx* on scopolamine-induced cognitively impaired mice's latency in the NW quadrant was also done. The results revealed that the latencies taken by mice which were administered with the methanolic stem bark extract of *L. eriocalyx*, at doses of 50 mg/kg bw and 200 mg/kg bw, were significantly longer than those of mice which received similar doses of the aqueous stem bark extract of the same plant (*p* < 0.05; Figure 15). However, there were no significant differences between the latencies in the NW quadrant that were recorded in mice, which received 100 mg/kg bw of the aqueous and the methanolic stem bark extracts of *L. eriocalyx* (*p* > 0.05; Figure 15).

### 4.3. Effects of the Aqueous and Methanolic Stem Bark Extracts of *L. eriocalyx* on *Ex Vivo* MDA Profiles in the Brains of Scopolamine-Induced Cognitively Impaired Mice Models

**Figure 16:**
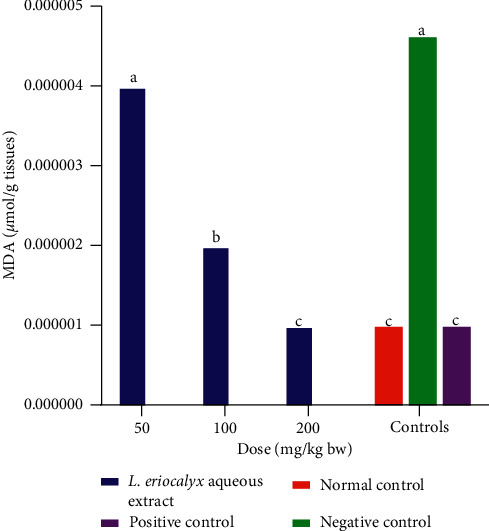
Effects of aqueous stem bark extracts of *L. eriocalyx* on MDA profile in the brains of scopolamine-induced cognitively impaired mice. Bars with the same letter are not significantly different (One-Way ANOVA followed by Fisher's LSD test; *p* > 0.05).

The MDA profile in the brains of the experimental mice administered with the aqueous stem bark extracts of *L. eriocalyx,* at a dose of 50 mg/kg bw, was not significantly different from the MDA profile in the brains of mice in the negative control group (*p* > 0.05; Figure 16). However, the MDA profiles in the brains of the mice that were administered with the *L. eriocalyx,* at doses of 100 mg/kg bw and 200 mg/kg bw, were significantly lower compared with the MDA profile in the brain of the mice in the negative control group (*p* < 0.05; Figure 16). Remarkably, the MDA profile in the brain of mice that received 200 mg/kg bw dose of *L. eriocalyx* was not significantly different from the MDA profiles in the brains of mice in the normal and positive control groups (*p* > 0.05; Figure 16).

**Figure 17:**
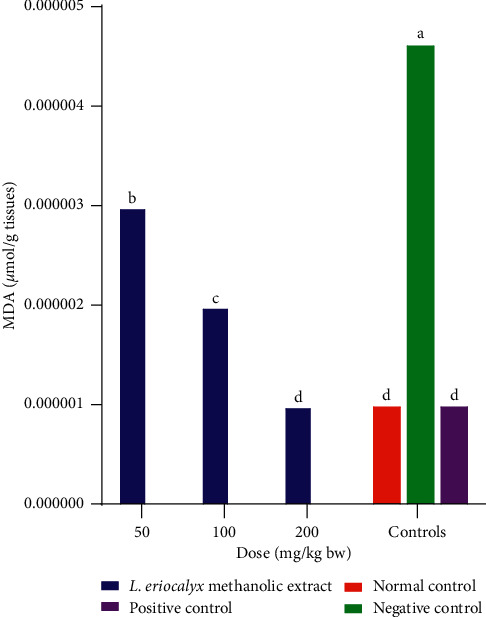
Effects of the methanolic stem bark extracts of *L. eriocalyx* on MDA profiles of scopolamine-induced cognitively impaired mice. Bars with the same letter are not significantly different (One-Way ANOVA followed by Fishers LSD test; *p* > 0.05).

On the other hand, the experimental mice that were treated with the methanolic stem bark extract of *L. eriocalyx,* at all the studied doses, had significantly lower MDA profiles in their brains compared with the MDA profile in the brains of mice in the negative control group (*p* < 0.05; Figure 17). A dose-dependent significant reduction in MDA profiles was observed in the brains of experimental mice, which received the methanolic stem bark extracts of *L. eriocalyx,* at all the three studied dose levels (*p* < 0.05). Remarkably, the MDA profile that was determined in the brain of mice which were treated with 200 mg/kg bw of the methanolic extract of *L. eriocalyx* was not significantly different from the MDA profiles in the brains of mice in the normal and positive control groups (*p* > 0.05; Figure 17).

**Figure 18:**
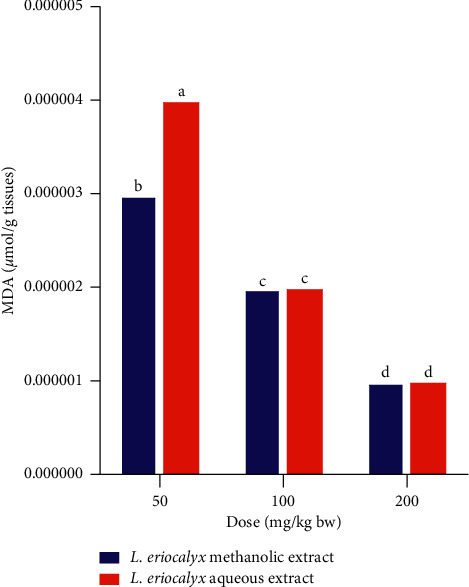
Comparison between the effects of the aqueous and methanolic stem bark extracts of *L. eriocalyx* on MDA profile in the brain of scopolamine-induced cognitively impaired mice. Bars with the same letter within the same dose level are not significantly different (unpaired Student's *t* test; *p* > 0.05).

Moreover, a comparison between the effects of methanolic and aqueous stem bark extracts of *L. eriocalyx* on MDA profiles was done. The experimental mice that were administered with the methanolic stem bark extract of *L. eriocalyx*, at a dose of 50 mg/kg bw, had significantly lower MDA levels in their brains compared with the MDA profile in the brains of mice that were treated with a similar dose of the aqueous stem bark extract of this plant (*p* < 0.05; Figure 18). However, at dose levels of 100 mg/kg bw and 200 mg/kg bw, there were no significant differences in MDA profiles in the brains of experimental mice treated with aqueous and methanolic stem bark extracts of *L. eriocalyx* (*p* > 0.05; Figure 18).

### 4.4. Qualitative Phytochemical Profiles of Studied Plant Extracts

Upon qualitative phytochemical screening of the aqueous and methanolic stem bark extracts *L. eriocalyx*, it was observed that anthracene glycosides and terpenoids were absent in all the studied plant extracts (Table 2). However, cardenolide glycosides, coumarins, phenols, steroids, saponins, and flavonoids were present in the aqueous and methanolic stem bark extracts of *L. eriocalyx* (Table 2). Additionally, alkaloids and tannins were present in all the studied plant extracts except in the methanolic stem bark extract of *L. eriocalyx*.

## 5. Discussion

Alzheimer's disease is the most common form of dementia, presenting progressive neurodegeneration and brain cell death [37]. Impaired cholinergic function presents various cognitive deficits, including difficulties in learning, memorizing events as well as reasoning/intelligence, observed in affected patients [38, 39]. Cognitive deficits are the hallmark features that are common in all types of dementia. Dementia is a briskly growing global public health problem, with a population of affected persons increasing by over 10 million to 88 million in the next ten years [3].

Various factors, including developmental abnormalities, ageing, diabetes mellitus, hypertension, obesity, genetic abnormalities, among others, can either initiate or exacerbate neurodegeneration culminating to cognitive deficits [3, 40, 41]. In current practice, various pharmacologic agents used to manage cognitive deficits and dementia are palliative, costly, and associated with adverse side effects [3, 17]. Based on this background, this study was designed to investigate the *in vivo* cognitive-enhancing, *ex vivo* antilipid peroxidation, and qualitative phytochemical profiles of the aqueous and methanolic stem bark extracts of *Lonchocarpus eriocalyx* (Harms.) as a potential alternative therapy for cognitive impairment and dementia.

In this study, the MWM method was adopted to evaluate the cognitive-enhancing effects of the aqueous and stem bark extracts of *L. eriocalyx* in scopolamine-induced cognitively impaired mice model. This technique has been broadly utilized to screen drug agents proposed for dementia and symptoms of cognitive impairment [42–46]. The MWM task is useful in assessing spatial learning and memory in laboratory animals and humans. Furthermore, it evaluates the integrity of the hippocampus, the brain region that modulates learning, memory, intelligence, among other cognitive functions [47]. In the MWM experiment, the rodent is allowed to swim around and navigate a pool of water to locate a hidden escape platform, after prior training, to evaluate its cognitive abilities [29].

Hyoscine hydrobromide (Scopolamine) is a muscarinic receptor antagonist that inhibits cholinergic transmission in both the peripheral and central nervous systems, resulting in cognitive impairment [48, 49]. This drug has extensively been used to induce dementia in animal models to evaluate the efficacy of drug agents in ameliorating dementia [45, 50, 51]. A drug capable of reversing or mitigating scopolamine effects is a potential antidementia agent. In the MWM task, the experimental animals that can locate the hidden escape platform within a short time, cover a short distance in the MWM task, and take longer latency in the target quadrant following treatment are appraised as cognitively intact and competent [52]. Therefore, the cognitive-enhancing drug agents should counteract/reverse the effects of scopolamine, in experimental models and translate to the shorter transfer latencies, shorter navigation distances, and longer latencies in the target quadrant [29, 35, 40, 42, 47, 52].

The results reported herein revealed that scopolamine successfully caused cognitive impairment in mice translating to longer transfer latencies and navigation distances in the negative control mice group. The shorter transfer latencies, shorter navigation distances, and longer latencies in the target quadrant recorded by the extract-treated mice were attributed to the cognitive-enhancing effects of these extracts. Overall, the methanolic stem bark extract of *L. eriocalyx* significantly enhanced spatial memory acquisition, retention, and memory in experimental mice than the aqueous extract. The results indicate that the methanolic extract contained higher concentrations of procognitive amalgams, which may have affected key cognitive processes and prevented brain damage. Partly, the cognitive-enhancing effects of the studied extracts of *L. eriocalyx* could be due to the bioactive principles they contain, which averted the effects of scopolamine, protecting against neural cell damage, thereby modulated hippocampal functioning [29, 40, 47, 52].

Moreover, a study by Rahimzadegan and Soodi [53] showed that scopolamine causes oxidative stress in the brain cells, especially in the hippocampus and cerebral cortex regions, causing amnesia, neurodegeneration, and apoptosis, as evidenced in persons who have Alzheimer's dementia [10, 54–56]. The cognitive-enhancing effects of the studied plant extracts can partly be attributed to the antioxidant properties which quench oxidative stress. Research has demonstrated that the brain is highly vulnerable to oxidative damage, which is caused by excessive free radicals [56]. This has been attributed to the increased metabolic activity of the brain, which calls for high oxygen utilization. Additionally, the presence of highly polyunsaturated fatty acids (PUFA) in higher degrees within the brain, which are easily damaged by oxidative stress, has been implicated. This is characterized by the weakened endogenous antioxidant defense, which drives brain damage, thereby resulting in impaired cognitive functioning [39, 57].

Lipid peroxidation is a well-known producer of cytotoxic components in the body as it causes lipid damage, especially those of biological membranes [39]. It has been implicated in many diseases and disorders, including those affecting the nervous tissue [58, 59]. Oxidative cell damage arising from lipid peroxidation proceeds through a feed-forward cascade of excessive free radical generation, thereby overwhelming the endogenous antioxidant mechanisms, leading to the production of toxic compounds comprising of aldehydes [59, 60]. Malondialdehyde (MDA) is the major aldehyde produced during oxidative stress-mediated lipid peroxidation in the body [39]. An increase in oxidative stress leads to increased damage to cellular components, which, in turn, result in elevated levels of MDA, which further modifies biomolecules to produce toxic amalgams [61].

Previous studies have implicated scopolamine as a potent inducer of oxidative stress leading to high MDA profiles [62–64]. The findings of this study collaborate well with those reported by Hritcu et al. [65], where high *ex vivo* MDA profiles in the brains of scopolamine-treated were recorded for the negative control group. Conversely, the normal control, positive control, and experimental group mice that were treated with the studied plant extracts demonstrated low MDA profiles. Notably, at doses of 100 mg/kg bw 200 mg/kg bw, both the aqueous and methanolic stem bark extracts of *L. eriocalyx* yielded significantly similar MDA profiles in mice, indicating that their active phytocompounds may have a similar mode of action in preventing either the production of the buildup of MDA in tissues. A reduction MDA profile by the aqueous and methanolic stem bark extracts of *L. eriocalyx* indicates successful oxidative stress attenuation and amelioration of cognitive deficits. Moreover, these findings suggest, in part, that the studied plant extracts contain antioxidant phytocompounds at different concentrations, which mitigated scopolamine-induced oxidative stress, reducing lipid peroxidation hence low MDA profiles.

Proper extraction is a crucial stage in the itinerary of medicinal plant processing for purposes of discovering bioactive compounds for drug development [52]. It is, therefore, imperative to employ a suitable extraction method to obtain desired soluble phytocompounds using appropriate solvents to obtain compounds with the desired activity [66].

According to La et al. [67], the method of extraction used determines the quantity and quality of the extracts obtained. Therefore, the extraction methods used in obtaining the stem bark extracts of *L. eriocalyx* were suitable. High yields are indicative of a high concentration of phytochemicals isolatable by that solvent. Conversely, low yields are attributable to the low concentration of phytochemicals and low extractive index. The low yields of the methanolic stem bark extracts of *L. eriocalyx* obtained in this study could be attributed to the low concentration of phytocompounds able to dissolve in this solvent. It is therefore suggestive that water, when used as a solvent of extraction, can capture polar phytocompounds better than methanol; however, the bioactivity of the extracted phytocompounds does not necessarily correlate with the yields of extracts.

The qualitative phytochemical profile of the aqueous and methanolic stem bark extracts of *L. eriocalyx* revealed the presence of various phytocompounds of pharmacologic significance. Research has determined that the environmental conditions at which medicinal plants grow play a key role in determining the composition and concentration of phytochemicals [64]. This can partly be explained by the fact that secondary metabolites are synthesized in response to stress to protect the plant [68].

Of the full range of plant phytochemicals, phenolic compounds demonstrate the broadest spectrum of pharmacologic bioactivity, which is attributable to their marked antioxidant effects [69]. It is therefore suggestive that the presence of phenols, coumarins, flavonoids, and tannins in the studied plant extracts ought to have potentially quenched oxidative stress, lowering MDA concentrations and ameliorating cognitive impairment in the brains of experimental mice [64]. Owing to the ethnomedical applications of *L. eriocalyx* herbal preparation in the management of cognitive deficits and cognitive-impairment-associated disorders like diabetes mellitus and hypertension in Kenya [22], this study demonstrates its potential therapeutic effects against cognitive impairment and dementia. Although the specific mechanism through which the studied plant extracts ameliorate cognitive deficits have not been elucidated, we propose modulation of the antioxidant system and the neuroprotective properties of polyphenolics as a probable mechanism.

## 6. Conclusions and Recommendations

The aqueous and methanolic stem bark extracts of *L. eriocalyx* have cognitive deficit ameliorating and MDA profile-lowering effects in Swiss albino mice. The studied plant extracts contain various phytochemicals of pharmacologic significance, including antioxidants. Based on the findings of this study, the aqueous and methanolic stem bark extracts of *L. eriocalyx* can be used for the management of cognitive impairment. Further studies targeting isolation and characterization of pure molecules from the studied plant extracts should be done. Moreover, the specific modes through which the studied plant extracts exert MDA profile-lowering and cognitive deficit ameliorating bioactivities should be elucidated.

## Figures and Tables

**Table 1:** Percentage of yields of studied plant extracts.

Plant	Percentage yield	
	Methanol extract	Aqueous extract
*L. eriocalyx*	9.17	16.21

**Table 2:** Qualitative phytochemical profiles of the aqueous and methanolic stem bark extracts of *L. eriocalyx*.

Phytochemical	Methanolic extract	Aqueous extract
Alkaloids	−	+
Cardenolide glycosides	+	+
Anthracene glycosides (anthraquinones)	−	−
Coumarins	+	+
Tannins	−	+
Terpenoids	−	−
Phenols	+	+
Steroids	+	+
Saponins	+	+
Flavonoids	+	+

+ = present; − = absent.

## Data Availability

All the data are included within the manuscript. Any additional information is available from the corresponding author upon request.
